# Supplementary material for: Hematological parameters in a population of male bakers exposed to high heat work environment
Source: PLoS One. 2022 Sep 16;17(9):e0274782. doi: 10.1371/journal.pone.0274782 (PMC9481027; doi:10.1371/journal.pone.0274782)
Supplement: S1 Appendix — (DOCX) [file pone.0274782.s001.docx]

**Appendix 1. Heat Exposure Questionnaire**

**Section 1: Demographic variables**

| 1. Age  …………………. years | 2. Nationality  .…………………… | | 3. Gender  1.Male 2. Female |
| --- | --- | --- | --- |
| 4. Weight  …………………Kg | 5. Height  ……………………. Cm | | 6. Education  1.Illiterate 2. Primary school 3. Secondary school or higher |
| 7. Marital Status   1. Single 2. Married 3. Divorced 4. Widower | | | 8. Income  ……………………US$ |
| 9. Job Title  ………………………………….. | | 10. Previous Occupations (if any) ………………………….. | |
| 11. Duration of continuous work in this Job  ……………………………………..years | | 12. Number of working hours  …..……….per day………………per week | |
| 13. Shiftwork  1. Day (7AM-4M) 2. Afternoon (2PM-10PM) 3. Night (10PM-6AM)  4. Other, specify…………………….. | | 14. Duration of shift  ……………………………………hours | |
| 15. Rest period during working shift, other than meal break   1. Yes 2. No   If yes, specify frequency of rest periods………………. minutes every………………..hours | | | |
| 16. Tobacco use status  1. Current 2. Ex-smoker 3. Never  If you specified that you are a current or an ex-smoker, please indicate type of tobacco use, average amount per day and for how many years…………………………….. ………………………………………………………………………………………….. | | | |

**Section 2: Questions related to heat exposure**

| 17. Recent or Past history of illness  1. Yes 2. No 3. Don’t Know  If yes, specify……………………………… | 18. Medications taken  1. Yes 2. No  If yes, specify………………………………. |
| --- | --- |
| 19. Drinking water or fluids while working  1. Yes 2. No  If yes, specify frequency: Once in every………………………………….minutes | |
| 20. Description of clothing  a. Vest: 1. With sleeves 2. No sleeves 3. None  b. Shirt: 1. Short sleeves 2. Long sleeves 3. None  c. Trouser: 1. Light 2. Medium 3. Heavy  d. Shorts: 1. Above knees 2. Below Knees 3. None  e. Briefs: 1. Yes 2. No  f. Socks: 1. Half 2. Long 3. None  g. Footwear: 1. Shoes 2. Boots 3. Sandals 4. None  h. Headwear 1. Yes 2. No  i. Others: 1. Yes (if yes , specify………………………………) 2. No | |
