# Supplementary material for: Hematological parameters in a population of male bakers exposed to high heat work environment
Source: PLoS One. 2022 Sep 16;17(9):e0274782. doi: 10.1371/journal.pone.0274782 (PMC9481027; doi:10.1371/journal.pone.0274782)
Supplement: Attachment — Submitted filename: Reply_template PLOS 2.docx [file pone.0274782.s002.docx]

**Re: PONE-D-21-23402**

**“Hematological Parameters in a Population of Male Bakers Exposed to Environmental Heat”**

**Reply to the editor and reviewers’ comments:**

| Reviewer Number | Original comments of the reviewer | Reply by the author | Changes done on page number and line number |
| --- | --- | --- | --- |
| Editor | Please ensure that your manuscript meets PLOS ONE's style requirements | Done |  |
| Editor | In your Methods section, please provide a justification for the sample size used in your study, including any relevant power calculations (if applicable). | There was no sample size calculation as all bakers in the area participated in the study.  The participant response rate was 100 percent | Page 3, line 73 |
| Editor | Provide additional information regarding how the participants were recruited for the study. | Already mentioned “Imam Abdulrahman bin Faisal University’s ethical review board (IRB-2018-03‐194) approved the study. The management of all bakeries in the study area were then contacted, and their cooperation requested. Written consent was obtained from all participants after explaining the aims of the study. Participants were told they were free to withdraw from the study at any time, and they were assured of the confidentiality of the recorded data”. | Page 5, line 108-112 |
| Editor | Finally please include additional information regarding the survey or questionnaire used in the study and ensure that you have provided sufficient details | Details on the questionnaire added.  Furthermore, copy of the questionnaire enclosed. | Page 4, lines 86-87  Page 15, Appendix 1 |
| Editor | Finally, the reviewers have identified that the language of the manuscript requires copy editing. Following the revision of the manuscript, we have concerns that the quality of language required further improvement. Please note that one of the publication criteria at PLOS ONE (https://journals.plos.org/plosone/s/criteria-for-publication#loc-5 )is that articles must be presented in an intelligible fashion and written in clear, correct, and unambiguous English. Please note that PLOS ONE cannot provide copyediting for manuscripts. | Done.  See attached editing certificate |  |
|  | | | |
| Reviewer #1 | Title: Please replace the “environmental heat” to “heat stress” or “high heat work environment” from the title. | Amended | Page 1, lines 1,2 |
| Reviewer #1 | Authors should preferably provide more information about the test population e.g. age group,  nationalities,  job experience, job role (baking, dough makers etc). | Amended  Amended  Amended | Page 5, lines 123-126  Page 6, lines 129-132  Page 6, lines 136-139 |
| Reviewer #1 | The hemoglobin (Hb) levels were found significantly different from control, but RBCs did not change. Please discuss in the discussion section. | Amended | Page 8, lines 192-196 |
| Reviewer #1 | Author mentioned in the result about difference in the WBC count between nationalities. This information should be given in a detailed tabulation form. | Table 3 added | Page 7, line 168  Page 14, Table 3 |
| Reviewer #1 | Table 2 shows that 84.6% of baker population have normal Hb. 13.4 % population have higher Hb levels. This is approximately 4% higher than control individuals having high Hb. What is the possible reason for high Hb in control population? | This was amended by the following statement: “……. which can be explained by sweating resulting in dehydration” | Page 8, lines 193-194 |
| Reviewer #1 | Did the author examined the 13.9% population having High Hb are smokers or have any medical condition?  Author also discussed less fluid intake is also related to low Hb levels in bakers. Author should consider all these factors while concluding the result. | We believe that this finding is not due to smoking as smoking was excluded as a confounding factor. Both bakers and control group were comparable in term of smoking  Already explained | Page 8, line 203-205 |
| Reviewer #1 | In the conclusion, the authors should provide the contribution of current study to the existing literature? | Conclusion amended | Page 9, lines 220-221 |
|  | | | |
| Reviewer #2 | 1. What is the necessity of addition of Ethnic origins, marital status, Income here? Please explain. | Already explained.  Therefore, all possible confounding factors related to the outcome were excluded. | Page 6, line 152-153 |
| Reviewer #2 | 2. In method section authors should include the inclusion and exclusion criteria. | Already explained | Page 3 Lines 73-74  Page 4, lines 75-84 |
| Reviewer #2 | 3. Authors at the beginning of Result section added Age – Income, which may be shifted to the method and if there is any found data that could be extracted and included in result. | Methods were amended.  Age and income were comparable among bakers and control and hence can be excluded as confounding factors | Page 4, line 86 |
| Reviewer #2 | 4. In conclusion, authors mentioned “abnormal hematological parameter”. Authors should mention the changes. Abnormal means? | Conclusion amended | Page 9, line 214 |
| Reviewer #2 | 5. In discussion authors added the explanation of RBC level changes – tissue damage. They may include the explanation of platelet change which is very significant. | Amended | Page 8, lines 200-202 |
|  | | | |
| Reviewer #3 | Author discussed that the blood samples were drawn after the work shift. It is important to mention the time of the sample drawn in a day i.e. morning/ noon/ evening and discuss the any possible effectiveness of circadian rhythm on the regulation of body temperature. | The venous blood sample were drawn after the work shift (morning /noon /evening).  A pattern of working long hours, including night and early morning shifts among the control group, to ensure comparability in this regard with bakers. Therefore, both the bakers and control are comparable in term of work shift | Page 4, lines 81-8 |
| Reviewer #3 | Author should discuss is there any correlation between increase hemoglobin label and decrease mean platelet volume. | Amended | Page 7, lines 169-170 |
| Reviewer #3 | Is there any differences of the body mass index between Bakers and controls? | Excellent point.  BMI was added. | Page 6, lines 147-151 |
