# Supplementary material for: Hematological parameters in a population of male bakers exposed to high heat work environment
Source: PLoS One. 2022 Sep 16;17(9):e0274782. doi: 10.1371/journal.pone.0274782 (PMC9481027; doi:10.1371/journal.pone.0274782)
Supplement: Attachment — Submitted filename: Reply_template PLOS final Aug 2022.docx [file pone.0274782.s003.docx]

**Re: PONE-D-22-06587**

**“Hematological Parameters in a Population of Male Bakers Exposed to High Heat Work Environment”**

**Reply to the editor and reviewers’ comments:**

| Reviewer Number | Original comments of the reviewer | Reply by the author | Changes done on page number and line number |
| --- | --- | --- | --- |
| Editor | Thank you for submitting your manuscript to PLOS ONE. After careful consideration, we feel that it has merit but does not fully meet PLOS ONE’s publication criteria as it currently stands. Therefore, we invite you to submit a revised version of the manuscript that addresses the points raised during the review process. | Thank you for inviting me to revise our manuscript. |  |
| Editor | In particular, reviewer 1's comment about other conditions influencing hematological parameters and their consideration in study design and analysis need to be addressed. | Done as explained below. |  |
| Editor | The discussion needs to reflect strengths and limitations of your study as well as a constructive discussion against the literature instead of just repeating results. | Limitations of our study was amended.  Discussion was amended. Our findings were contrasted with previous published studies. | Page 9, lines 236-238  Pages 8 and 9 |
| Editor | Please also address the reviewer comments about the applicability of an English questionnaire among workers of whom 22% were illiterate. | Done as explained below. |  |
| Editor | Please explain your calculation of the response proportion (eligible subjects, contacted subjects, participants), because 100% response among eligible subjects appears highly unlikely. | Amended | Page 4, lines 78-79 |
|  | Please also check the p-value for the comparison of smokers (p=0.198), which appears questionable, given that 63.5% of bakers were smokers compared to 52% of controls. | We double checked the p-value in this regard, and it was statistically insignificant.  This was also supported by the statement “The mean number of cigarettes smoked per day among the bakers was 9.9 (SD: 10.9), compared to 10.8 (SD: 12.7) among the controls (P=0.544).” | Page 6, lines 150-151 |
|  | | | |
| Reviewer #1 | 1. Statements in result of the abstract needs a p value. | Abstract amended | Page 2, lines 40 and 42 |
| Reviewer #1 | 2. Rationale of the study is not properly mentioned. | Amended | Page 3, page 74-75 |
| Reviewer #1 | 3. Design of the study is the greatest flaw. Exclusion criteria is not clear. Many conditions may affect blood hemogram parameters. Were they excluded or not? | Excellent point.  Exclusion criteria were already mentioned.  In the questionnaire we asked the bakers: Q 17. Recent or Past history of illness?  Q 18. Medications taken  The following statement” All bakers in this study worked for more than one year in the bakeries and were healthy and had no history of medical conditions that may affect blood hemogram parameters”. Added | Page 4, lines 80-81  Page 7, lines 161-162 |
| Reviewer #1 | 4. Statistics were not expressed properly. For instance, it is unclear from the text which normality test was applied to the study variables. | Normality test was mentioned in Table 2 |  |
| Reviewer #1 | 5. Presentation of results is in a form that is difficult to understand. | The results section has been modified to reflect further clarity. Thank you. | Page 5-8 |
| Reviewer #1 | 6. Discussion is just a repetition of the results rather than being a constructive discussion of them along with literature data. | Amended. There were few published studies related to our findings, however relevant literatures were quoted. | Page 8 and 9 |
| Reviewer #1 | 7. Inappropriate self-citations were noted. | As stated above, there were few medical literatures related to this topic. Most of the journals accept self-citations. |  |
|  | | | |
| Reviewer #2 | I understood that the participating bakers had significantly higher Hb-levels and lower platelet-counts than the control-group. These findings are associated with significantly higher WGB-temperatures of the bakers work environment and lower rehydration than in the control group. These are your main findings as far as I understood. But within the "Results"-section these findings are hidden behind other data like marital status, nationality and level of education. Why? | We believe that marital status, nationality and level of education were confounding factors related to the outcome and these should be excluded. We found that both the bakers and the control group were considered comparable in terms of the assessed confounding factors. |  |
| Reviewer #2 | Wouldn't it be better to summarize age and BMI in a table with the statement that groups are comparable and then to present your central findings? | These findings were explained in the text. | Page 6, lines 154-155  Page 7, lines 156-158 |
| Reviewer #2 | I do not understand the benefits or scientific value of a comparison of e.g., marital status, nationality, income and level of education. | As explained above: These were confounding factors related to the outcome and should be excluded. We found that both the bakers and the control group were considered comparable in terms of the assessed confounding factors. |  |
| Reviewer #2 | The level of illiterates off 22.4% could devalue your work because readers could interpret this as more than a fifth of your bakers were not able to fill in your questionnaire in their own language. Not to speak of English language. | Excellent point.  Amended. | Page 4, line 95 |
| Reviewer #2 | By the way: I don't think it is appropriate to have sentences like "It is believed that (...)" with a reference in the "Results"-section. This belongs to the "Discussion"-section. | Amended and moved under discussion | Page 8, lines 192-195 |
| Reviewer #2 | Further I do not understand what was your hypothesis. Had there been a power calculation with the pilot study you mentioned or other scientific work?  Please take into account that usually it is not appropriate to collect data and then test "wildly" for statistical differences. You should articulate a hypothesis generated with preceding findings of yours or others and then test for it. | Hypothesis added | Page 3, line 72-73 |
| Reviewer #2 | In the "discussion"-section you write: "...reductions in RBC and WBC have been reported in animal studies after exposure to high heat, and this was explained as being caused by tissue damage that was dependent on the level and duration of the heat exposure." But your data does not support this! And animal studies might be problematic in this particular topic, because humans are not fish and they usually do not have fur.  It might be useful to concentrate at the Hb instead: If Hb increases and RBC stays almost the same: Might it be, that not only the plasma volume is shrinking but the volume of the erythrocytes too? Is it really tissue damage or might it be, that this is a physiological response to survive extreme conditions? E.g., might the reduction of platelets be a protective mechanism to circumvent higher blood viscosity and thromboembolic events? | Thank you for pointing this out.  This paragraph was amended and your thoughts in this regard were added. | Page 8, lines 206-210  Page 9, line 211 |
| Reviewer #2 | Could you provide data of the amount of fluids the participants were drinking? I think this might be more relevant than the time/frequency they were drinking. | Unfortunately, we did not measure the amount of fluid intake, which was the appropriate way to do. Instead, the data related to fluids intake were collected by means of questionnaire and we believe it was a surrogate of the amount of fluid intake. |  |
| Reviewer #2 | Could you make a subgroup analysis with bakers with the hottest working conditions compared with the less hot working conditions? And the same with high and low fluid intake during the shift, respectively? | Excellent point.  This was beyond the scope of our study |  |
| Reviewer #2 | I couldn't develop any feeling about the real work environment of the bakers: Was it hot & dry? Or more humid? The WBGT is not telling that. Could you provide additional data about humidity, because this significantly alters the ability of the human body to adopt to high temperatures? | The statement “The relative humidity was read off the psychometric chart. The average relative humidity in bakeries was 27%±15%. “ added | Page 5, lines 105-106 |
| Reviewer #2 | At scientific papers like PLOS usually it is not appropriate to cite whole medical textbooks. If there is a scientific finding you cannot find anywhere else (primary scientific paper), you should at least provide the exact page(s) where the fact could be found. | Reference 1 was updated. | Page 11 |
| Reviewer #2 | Then there is another point in your data which is not self-explanatory: Why the bakers are so young and why is their working experience so low? Do they drop out their jobs because of the high physical demands or because they earn enough money to go back to their home countries or do they get promoted to higher positions in their bakeries without having these working conditions? | We have no explanation, why were the bakers so young? However, both (the bakers and control) are comparable in term of age and this was excluded as a confounding factor |  |
|  | | | |
| Reviewer #3 | Regarding the statistically significant difference in hemoglobin and platelet values, the authors need to explain how such findings are compatible with the literature on exposure to heat stress, since terms such as "sweating resulting in dehydration" or "tissue damage" are very unspecific to explain the physiological process. Mainly considering that there was no difference on white or red cells. | Discussion in this regard was amended | Page 8, lines 206-210 |
| Reviewer #3 | And Why hematocrit data was not presented, which is directly related to dehydration? | Thank you for point this out.  Hematocrit was not investigated in this study. |  |
| Reviewer #3 | I suggest to include a table with the analysis about data collected, specially regarding work characteristics - such as working hours and exposure time, which are crucial to understanding the impact of occupational hazards. | This already explained in the manuscript | Page 7, line 182  Page 8, lines 183-184 |
| Reviewer #3 | The authors chose to present only the significant difference in the nationality of the participants, but they do not present a discussion for this finding - was it a random result? | Excellent point.  Yes, it was a random result, and we did not find literature to explain this phenomenon |  |
| Reviewer #3 | Finally, the study could present more information about exposure to heat stress evaluation, as there is only the report of the average among exposed and controls. Maybe the details of the range of temperature variation can be more explored in discussion about excessive exposure. | Thank you for pointing this out.  We already mentioned that WBGT were taken simultaneously at the different locations at which the workers performed their routine job duties. We felt that the average WBGT was more appropriate to report. | Page 5, lines 104-105 |
