# Supplementary material for: Hematological parameters in a population of male bakers exposed to high heat work environment
Source: PLoS One. 2022 Sep 16;17(9):e0274782. doi: 10.1371/journal.pone.0274782 (PMC9481027; doi:10.1371/journal.pone.0274782)
Supplement: Attachment — Submitted filename: Response to Reviwers September 2022.docx [file pone.0274782.s004.docx]

**Re: PONE-D-22-06587R1**

**“Hematological Parameters in a Population of Male Bakers Exposed to High Heat Work Environment”**

**Reply to the editor and reviewers’ comments:**

| Reviewer Number | Original comments of the reviewer | Reply by the author | Changes done on page number and line number |
| --- | --- | --- | --- |
| Editor | Please review your reference list to ensure that it is complete and correct. | Done.  References checked and updated, and changes highlighted in yellow | Pages 12-15 |
| Editor | In particular, please refer again to Reviewer 1's original comments that need to be considered in more detail.  As Reviewer 3 has noted, the limitations section should focus more on potential biases, such as recruitment, non-response, exposure assessment, and statistical analysis. | Done as explained below. |  |
|  | | | |
| Reviewer #1 | Reviewer #1: Authors somewhat improved the quality of the paper in revised version. However significant proportion of my criticisms were not addressed adequately. | Thank you.  Please note our details response as outlined below. |  |
| Reviewer #1 | 1. Statements in result of the abstract needs a p value. | Abstract amended. | Page 2, lines 40 and 42 |
| Reviewer #1 | 2. Rationale of the study is not properly mentioned. | Now added. | Page 3- and 4-lines 74-77 |
| Reviewer #1 | 3. Design of the study is the greatest flaw. Exclusion criteria is not clear. Many conditions may affect blood hemogram parameters. Were they excluded or not? | Design of the study, Inclusion and exclusion criteria now explained in detail.  Also, the findings of this explained. | Page 4, lines 81-88  Page 6, lines 132-133 |
| Reviewer #1 | 4. Statistics were not expressed properly. For instance, it is unclear from the text which normality test was applied to the study variables. | Normal range was mentioned in Table 2 | Table 2, page 16 |
| Reviewer #1 | 5. Presentation of results is in a form that is difficult to understand. | The results section has been modified to reflect further clarity. | Page 6-8 |
| Reviewer #1 | 6. Discussion is just a repetition of the results rather than being a constructive discussion of them along with literature data. | Amended. | Page 8 and 9 |
| Reviewer #1 | 7. Inappropriate self-citations were noted. | Self-citations (references 13 and 14) were deleted and replaced with relevant ones. | Page 13, lines 331-336 |
|  | | | |
| Reviewer #3 | Maybe limitations session should be focused in biases, such as recruitment, losses, exposure, evaluation, analyze. | Limitations section amended. | Page, 10 lines 250-251 |
